# Formation of gadolinium–ferritin from clinical magnetic resonance contrast agents†

**DOI:** 10.1039/c9na00567f

**Published:** 2020-08-31

**Authors:** Jitka Neburkova, Aaron M. Rulseh, Shery L. Y. Chang, Helena Raabova, Jana Vejpravova, Martin Dracinsky, Jan Tarabek, Jan Kotek, Mohan Pingle, Pavel Majer, Josef Vymazal, Petr Cigler

**Affiliations:** Institute of Organic Chemistry and Biochemistry of the CAS Flemingovo nam. 2 166 10 Prague Czechia cigler@uochb.cas.cz; Department of Radiology, Na Homolce Hospital Roentgenova 2 150 30 Prague Czechia josef.vymazal@volny.cz; Electron Microscopy Unit, Mark Wainwright Analytical Centre, and School of Materials Science and Engineering, University of New South Wales Sydney NSW 2052 Australia; Department of Inorganic Chemistry, Faculty of Science, Charles University Hlavova 8 128 43 Prague 2 Czechia; Department of Condensed Matter Physics, Faculty of Mathematics and Physics, Charles University Ke Karlovu 5 121 16 Prague 2 Czechia

## Abstract

Gadolinium deposition in the brain following administration of gadolinium-based contrast agents (GBCAs) has led to health concerns. We show that some clinical GBCAs form Gd^3+^–ferritin nanoparticles at (sub)nanomolar concentrations of Gd^3+^ under physiological conditions. We describe their structure at atomic resolution and discuss potential relevance for clinical MRI.

## Abstract 2

We show that clinical gadolinium-based contrast agents can form Gd^3+^-ferritin nanoparticles under physiological conditions. We suggest they can contribute to the T_1_ intensity changes in ferritin-rich brain areas observed in patients examined by MRI.

## Graphical abstract

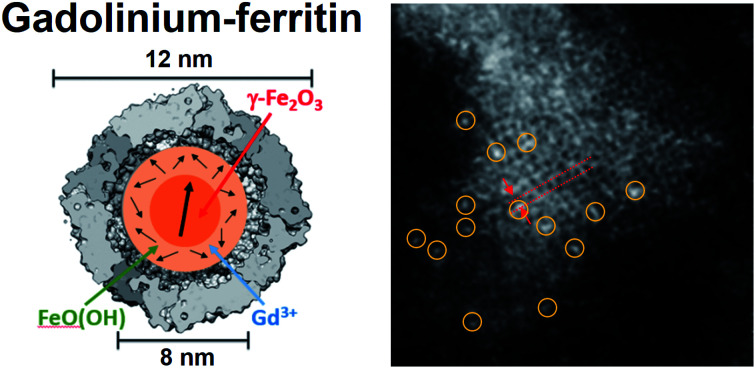

GBCAs have been applied in clinical magnetic resonance imaging (MRI) for 30+ years, serving as an indispensable component of roughly 30 million procedures annually.^1^ GBCAs contain Gd^3+^ complexed with linear or macrocyclic polydentate amino-carboxylate ligands. Due to the toxicity of free Gd^3+^,^2^ the stability of GBCAs has important safety implications. Recently, there has been increasing interest in gadolinium deposition in the brain following repeated GBCA application,^3–5^ especially with some linear agents.^6,7^ Histochemical confirmation of residual gadolinium in the dentate nucleus and globus pallidus^8^ reinvigorated discussion about the safety of GBCAs.^9–15^ Despite the generally recognized *T*_1_-weighted signal intensity increase in these brain regions after repeated GBCA application, both the mechanism and chemical form responsible for this effect remain unknown.^16–20^ In rat brains, three main forms of Gd^3+^ were identified after GBCA administration: inorganic insoluble forms with no relevant relaxivity, original small-molecule GBCAs, and a macromolecular form.^21^ We hypothesized that a complex of Gd^3+^ and the metalloprotein ferritin may contribute to the preferential Gd deposition in the dentate nucleus and globus pallidus^8^ and to the observed signal intensity changes in MRI.

We based this hypothesis on the following considerations. First, the highest concentration of non-haem iron in brain is found in the globus pallidus (21 mg Fe per 100 g fresh weight).^22^ Non-haem iron in the brain is mainly located in the ferritin form.^23^ Further, ferritin is able to effectively capture ions other than Fe^3+^*in vivo*,^24^ as demonstrated by isolation of an Al^3+^–ferritin complex from the human brain, horse spleen and liver.^25^ Attachment of Gd^3+^ in an ionic or chelated form to macromolecular or nanoparticulate structures including ferritin^26,27^ greatly enhances *T*_1_ relaxivity. Finally, soluble macromolecular Fe- and Gd-rich fractions isolated from a rat brain had molecular weights higher than 250–300 kDa.^21^ Ferritin (830 kDa) can be considered as a plausible component of this fraction.

Here, we show that free Gd^3+^ (aqua)ions present at (sub)nanomolar equilibrium concentrations in GBCA solutions bind unexpectedly strongly to the ferrihydrite core of ferritin under physiologically relevant conditions. We mapped the presence of individual Gd^3+^ ions in the cores using high-angle annular dark field scanning transmission electron microscopy (HAADF-STEM) and addressed Gd^3+^ incorporation using magnetic measurements conducted in the context of extended core–shell and modified two-component models.^28,29^ We quantitatively examined the interactions of six clinically used GBCAs with ferritin and assessed the role of structural, thermodynamic and kinetic parameters of the GBCAs in the formation of Gd^3+^–ferritin nanoparticles. We also investigated the role of the ferritin type (equine *vs.* human) and the impact of human serum proteins on the binding of Gd^3+^ to ferritin. Finally, we analyzed the relaxation properties of the new Gd^3+^–ferritin metalloprotein.

Ferritin is an iron storage protein which is able to accumulate and store up to 4500 atoms of iron. Ferritin nanoparticles have an outer diameter of approximately 12 nm and an inner cavity of 7–8 nm. The protein shell consists of 24 subunits which self-assemble in dimers and form a dodecameric cage around a ferric oxyhydroxide core.^30^ To obtain representative Gd^3+^–ferritin nanoparticles, we incubated linear GBCA 1 (Fig. 1) with equine ferritin under physiologically relevant conditions (pH 7.4, 0.9% NaCl, 37 °C) and purified the resulting species by dialysis. Using HAADF-STEM imaging, we compared its structure and morphology with those of ferritin.

**Fig. 1:**
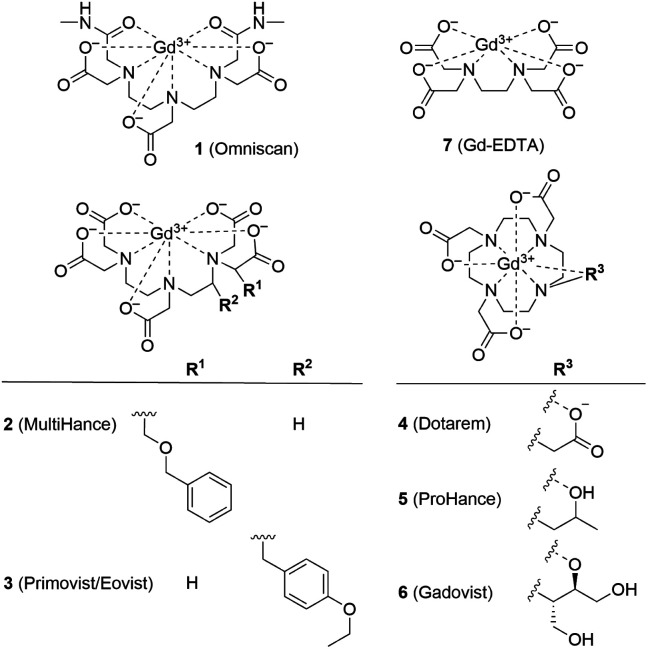
Structural formulas of GBCAs. The water molecule coordinated to the central Gd^3+^ ion is omitted for clarity.

The image intensity in HAADF-STEM is approximately proportional to *Z*^2^, where *Z* is the atomic number. The ferritin protein shell, consisting of elements with low *Z*, is nearly invisible; the bright contrast in Fig. 2a and d comes from characteristic mono-dispersed, 8 nm iron oxyhydroxide nanoparticles of ferritin,^31,32^ revealing the ferrihydrite structure at atomic-resolution (Fig. 2b, e, S1 and S2†).^33^ In contrast to ferritin, the Gd^3+^–ferritin nanoparticles possess sites with unusually bright spots corresponding to Gd atoms, which can be quantified in line scans along the lattice lines (Fig. 2f). The intensities cannot be attributed to Fe atoms alone, as this would indicate an “atomically spiky” core, which is unlikely. The position of the Gd bright spots (Fig. 2e) suggests that Gd atoms are likely to be present in substitutional positions, as they mostly coincide with the atomic columns of ferrihydrite. We did not observe Gd in the protein shells nor in control samples of apoferritin incubated with 1 (Fig. S3 and Table S1†).

**Fig. 2:**
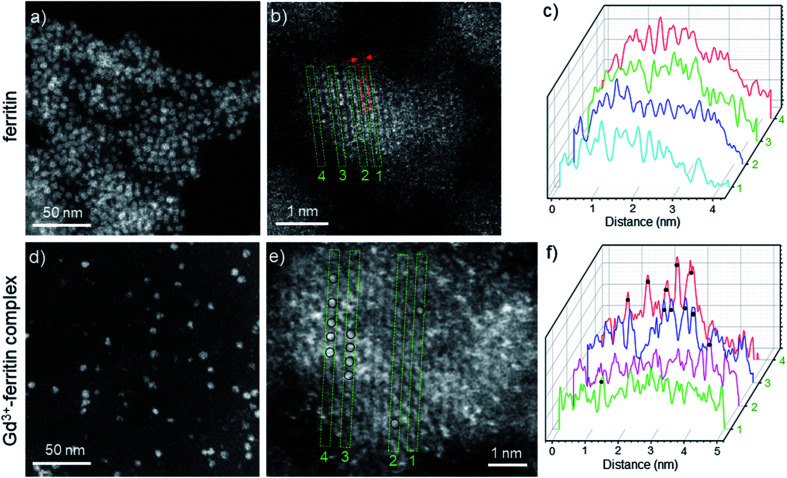
HAADF-STEM images of Gd-free ferritin (a–c) and Gd^3+^–ferritin nanoparticles (d and e). Images show the typical areas of mono-dispersed ferritin (a) and Gd^3+^–ferritin (d). Atomic resolution images of ferritin (b) and Gd^3+^–ferritin nanoparticles (e) the ferrihydrite phase with a characteristic lattice spacing of 2.59 ± 0.07 Å.^21^ Based on our electron scattering simulations, the intensity of Gd is approximately 6-fold higher than that of Fe in the cores. Selected line scans 1–4 across the atomic columns for ferritin (c) and Gd^3+^–ferritin (f) particles correspond to the green rectangles 1–4 in (b) and (e), respectively. The sites containing Gd atoms are marked with black circles.

To determine the spatial location of Gd^3+^ inside the oxyhydroxide phase, we studied ferritin and Gd^3+^–ferritin using superconducting quantum interference device (SQUID) magnetometry. Compared to ferritin, Gd^3+^–ferritin had a significant paramagnetic tail in the zero field-cooled curve and field-cooled curves at low temperatures, a moderate increase in the mean blocking temperature, and enhanced paramagnetic contribution in the magnetization isotherm (see the ESI† for additional discussion). These results show that Gd^3+^ does not enter deep into the ferritin core and is most likely incorporated in the outermost shell of the core. This observation is corroborated by the ∼8% enhancement in effective magnetic anisotropy and the invariant magnitude of the mean magnetic moment (size) of the maghemite component.

Gd^3+^ incorporation into ferritin was also confirmed by electron paramagnetic resonance (EPR) at 77 K. Consistent with the results from HAADF-STEM and SQUID magnetometry, we observed a change in ligand field/symmetry of Gd^3+^ after transfer from 1 to the oxyhydroxide (ferritin) core, indicated by a *g*-factor difference of ∼0.05 in the low-field region (Fig. S5 and additional discussion in the ESI†).

Overall, our data unambiguously support effective binding of nanomolar concentrations of free Gd^3+^ ions released from GBCAs to the iron oxyhydroxide core inside ferritin capsules. This is in agreement with previous work showing formation of stable Gd^3+^ oxyhydroxide from free Gd^3+^ inside apoferritin at pH 6.5.^34^ Other possible binding scenarios, such as inclusion of GBCAs inside the protein shell^26,35^ and binding/adsorption of free or chelated Gd^3+^ to the protein shell,^36^ clearly did not contribute to formation of Gd^3+^–ferritin species under our conditions (Table S1†). The structure of the protein shell also corroborates this conclusion, because the access to the cavity is limited by channels (diameter ∼ 0.4 nm) formed at intersections of protein subunits.^30^ While ions and water can diffuse through the channels, GBCAs with a typical diameter of 0.8 nm or larger cannot enter the ferritin interior under physiological conditions.^26^

To simulate the conditions occurring in the body, we used 1.43 mM GBCAs, reflecting their approximate concentration in blood after intravenous injection. After 24 h, Gd^3+^ bound to ferritin was not in thermodynamic equilibrium with GBCAs. However, the chosen conditions enabled us to distinguish the ability of GBCAs to interact with ferritin under physiological conditions. Because the biological half-life of GBCAs is in the order of hours,^16^ we surmised that these conditions may approximate the situation (timescale and concentration) during the interaction of GBCAs with ferritin in a patient’s brain.

We analyzed the composition of Gd^3+^–ferritin nanoparticles formed upon interaction with a representative set of clinical GBCAs (1–6) and with non-clinical chelate 7 (Fig. 1). The Gd^3+^ loadings were strikingly different for the various GBCAs, spanning a 4-order-of-magnitude range from a negligible interaction (4 and 5) to 7.2 and 46 Gd^3+^ ions per ferritin molecule for 1 and 7, respectively (Fig. 3). Gd concentrations were negligible in control GBCA solutions dialyzed in the absence of ferritin. Alternatively, we also tested purification (*i.e.* removal of the excess of 1) by gel permeation chromatography which provided identical results (see Table S2 and additional discussion in the ESI†).

**Fig. 3:**
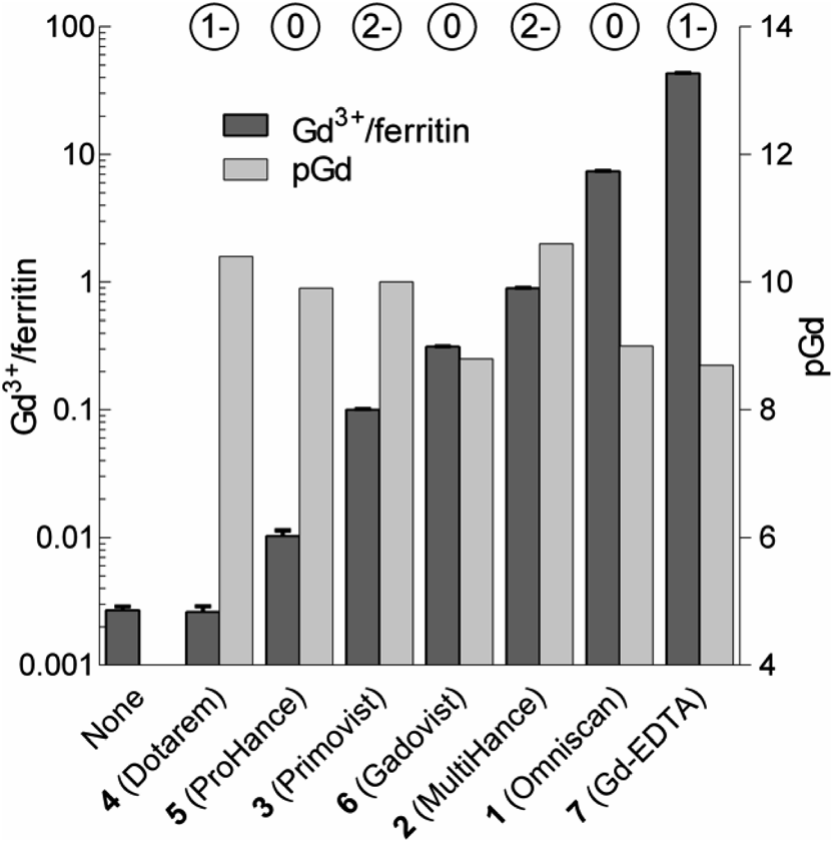
Loadings of Gd expressed as the number of Gd^3+^ ions per one ferritin nanoparticle (Gd^3+^/ferritin) formed in the presence of 1–7. “None” denotes a control ferritin sample incubated without GBCAs. The error bars represent standard deviations from three measurements. pGd values in GBCA solutions were calculated for the incubation conditions (see the ESI†). The circled numbers above the columns show the overall charge of the GBCAs at pH 7.4.

In a separate incubation experiment with 1 we addressed potentially different behavior of the equine and human ferritin. We used a different batch of equine ferritin; nevertheless, we obtained similar Gd^3+^ loadings to the previous experiment (11.3 Gd^3+^ ions per ferritin molecule compared to the previous value 7.2). The Gd^3+^ loading of human ferritin reached a similar level (8.6 Gd^3+^ ions per ferritin; Table S3†), which suggests a similar binding capacity for both ferritins. We also tested the impact of serum proteins on the binding equilibrium, because proteins relevant to iron metabolism such as transferrin can potentially influence the Gd^3+^ speciation. We incubated both equine and human ferritins with 1 in human serum. The presence of the serum proteins led to a decrease in total Gd^3+^ loads by a factor of ∼3. We obtained 2.7 Gd^3+^ ions per human ferritin formed in serum compared to 8.6 in the buffer. Similarly, equine ferritin bound 3.7 Gd^3+^ ions per ferritin in serum compared to 11.3 in the buffer. Regardless of the lower total Gd^3+^ loads achieved in the presence of serum proteins, the experiments confirmed the formation of Gd^3+^–ferritin in both tested environments (human serum or buffer) and ferritin types (equine or human).

Considering that chelate stability is a crucial factor in deposition of gadolinium in the brain,^37^ we tested whether thermodynamic stability of the chelates may be responsible for these striking differences between GBCAs. To objectivize metal binding by various ligands, we calculated the concentrations of free Gd^3+^ (aqua)ions (pGd = −log[Gd^3+^]) in solutions of 1–7 under our incubation conditions (Fig. 3 and Table S4†).

Neither pGd values (corresponding to GBCA thermodynamic stability) nor chelate charges correlated with the observed Gd^3+^–ferritin loadings (Fig. 3 and Table S5†). Even 7 showed comparable thermodynamic stability to some of the clinically used GBCAs, although 7 is known to be toxic.^38^ The toxicity of 7 has been attributed to the low selectivity of EDTA for Gd^3+^ over biogenic metals such as Ca^2+^, Cu^2+^, and Zn^2+^, the presence of which supports the release of free Gd^3+^.^39^ However, no additional metal ion was present in our experiments, and thus this explanation does not apply. Therefore, we next tested whether Gd^3+^ loadings to ferritin is driven by the kinetic lability of a given GBCA. Available kinetic data showed general trends fully consistent with the order of complex lability (see the ESI†). For instance, the great propensity of 1 to form Gd^3+^–ferritin complexes is consistent with the fact that nephrogenic systemic fibrosis was almost exclusively connected to the application of 1.^40^ Moreover, the kinetic inertness of linear 2 and 3 is much higher compared to that of other linear chelates^41,42^ and approaches that of macrocyclic complexes,^43^ in agreement with our experimental data. This result is also relevant to the current critical discussion on schematic sorting the biological behavior of GBCAs according to their linear or macrocyclic character.^11,44–47^ This simplistic differentiation is apparently not in agreement with our results, suggesting that the impact of Gd^3+^ released from each GBCA in a biological environment has to be evaluated individually.

Finally, we focused on quantitative comparison of the effects of the presence of Gd^3+^ in ferritin on relaxation properties relevant for MRI. We measured *T*_1_, *T*_2_, and 
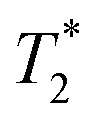
 relaxation times for aqueous solutions containing Gd^3+^–ferritin nanoparticles prepared from different GBCAs. The nanoparticles with high Gd^3+^ loads (Fig. 3) showed consistently shortened relaxation times (Table 1). *T*_1_ and *T*_2_ shortening was significant for both kinetically labile 1 (7.2 Gd^3+^/ferritin) and 7 (43 Gd^3+^/ferritin). For kinetically stable chelates 6 and 2, the shortening was negligible or insignificant, respectively.

**Table 1:** *T*_1_, *T*_2_, and 
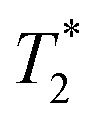
 relaxation times of Gd^3+^–ferritin solutions prepared from different GBCAs (1.00 mg mL^−1^ ferritin in HEPES, pH 7.4)

	*T*_1_ [s]	*T*_2_ [ms]	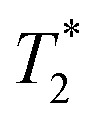 [ms]
Buffer	2.83 ± 0.05	510 ± 40	99
Ferritin	1.87 ± 0.02	58.6 ± 0.2	30
Ferritin + 6	1.80 ± 0.05	59.1 ± 1.1	30
Ferritin + 2	1.73 ± 0.01	54.7 ± 0.3	27
Ferritin + 1	1.70 ± 0.04	49.5 ± 0.3	25
Ferritin + 7	0.80 ± 0.01	38.9 ± 0.1	21

Thanks to their size and geometry, the ferritin nanoparticles have the potential to change the relaxation properties of the encapsulated Gd^3+^ ions, leading to increased *T*_1_-weighted contrast.^26,34,35^ Our data show that Gd^3+^–ferritin nanoparticles with very low Gd^3+^ loads can cause a significant change in relaxation properties.

## Conclusions

We identified that Gd^3+^–ferritin nanoparticles unexpectedly form at remarkably low, (sub)nanomolar concentrations of Gd^3+^ released from GBCAs under physiologically relevant conditions (pH 7.4, 0.9% NaCl, 37 °C). Gd^3+^–ferritin also forms in human serum from equine and human ferritin. The structure of the nanoparticles at atomic resolution shows that Gd^3+^ ions bind to the surface region of the oxyhydroxide core of ferritin. The Gd^3+^ loading does not depend on thermodynamic stability and is driven by the kinetic lability of a given GBCA. We also provide evidence of the significant influence of Gd^3+^–ferritin on *T*_1_, *T*_2_, and 
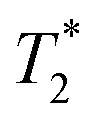
 relaxation times.

Because binding of Gd^3+^ released from GBCAs to ferritin was significant under physiologically relevant conditions (human serum), we suggest that formation of Gd^3+^–ferritin can contribute to the *T*_1_ intensity changes in ferritin-rich brain areas observed in patients after repeated GBCA application. Naturally, our work does not exclude the possibility that Gd^3+^ can interact *in vivo* with other macromolecular or nanoparticulate species. We are also aware of the potentially different properties of particular types of ferritin. According to the tissue function, ferritins differ in ratio of heavy and light chains and also in the iron load. The heavy chain, which participates in the oxidation of Fe^2+^ to Fe^3+^, is more prevalent in tissues with rapid iron uptake and release (such as the brain, heart and, kidneys). The light chain, which supports mineralization of iron and the formation of the ferritin iron core, is present mainly in tissues with long-term iron storage (the liver and spleen).^48–50^ Moreover, the heavy/light chain ratio varies in the different regions of the brain^24^ and depends also on the cellular localization (nuclear *vs.* cytoplasmic).^51^ In this study, we used equine spleen and human liver ferritins which may differ from the brain ferritin in Gd^3+^ complexation rates and the resulting Gd^3+^ loads. Clearly, an *in vivo* study would be beneficial to further support our observations.

In conclusion, we believe that our data would bring a new viewpoint to this medicinally relevant issue and will stimulate further studies leading to uncovering molecular aspects of Gd^3+^ deposition in the human body.

## Conflicts of interest

J. V. is a consultant for Novocure Inc. and Bracco Inc.

## Supplementary Material

NA-002-C9NA00567F-s001
