# Supplementary material for: Formation of gadolinium–ferritin from clinical magnetic resonance contrast agents
Source: Nanoscale Adv. 2020 Aug 31;2(12):5567–71. doi: 10.1039/c9na00567f (PMC9417687; doi:10.1039/c9na00567f)
Supplement: NA-002-C9NA00567F-s001 [file NA-002-C9NA00567F-s001.pdf]

Electronic Supplementary Material (ESI) for Nanoscale Advances.
This journal is © The Royal Society of Chemistry 2020

Electronic Supplementary Information: Formation of gadolinium-ferritin from clinical magnetic resonance contrast agents

Jitka Neburkova,[a] Aaron M. Rulseh,[b] Shery L. Y. Chang,[c] Helena Raabova,[a] Jana Vejpravova,[d,e] Martin Dracinsky,[a] Jan Tarabek,[a] Jan Kotek,[d] Mohan Pingle,[b] Pavel Majer,[a] Josef Vymazal*[b] and Petr Cigler*[a]

[a.] Institute of Organic Chemistry and Biochemistry of the CAS, Flemingovo nam. 2, 166 10, Prague, Czechia
[b.] Department of Radiology, Na Homolce Hospital, Roentgenova 2, 150 30, Prague, Czechia
[c.] Eyring Materials Center and School of Molecular Science, Arizona State University, Tempe, AZ 85284, USA
[d.] Department of Inorganic Chemistry, Faculty of Science, Charles University, Hlavova 8, 128 43 Prague 2, Czechia
[e.] Department of Condensed Matter Physics, Faculty of Mathematics and Physics, Charles University, Ke Karlovu 5, 121 16 Prague 2, Czechia
*Corresponding authors: cigler@uochb.cas.cz, josef.vymazal@volny.cz

Experimental Procedures

GBCAs
The following six commercial GBCAs were used: **1** (gadodiamide; Omniscan; GE Healthcare, Little Chalfont, Buckinghamshire, UK), **2** (gadobenate dimeglumine; MultiHance; Bracco, Milan, Italy), **3** (gadoxetate disodium; Primovist/Eovist; Bayer Schering Pharma AG, Berlin, Germany), **4** (gadoterate meglumine; Dotarem; Guerbet, Villepinte, France), **5** (gadoteridol; ProHance; Bracco, Milan, Italy), and **6** (gadobutrol; Gadovist; Bayer Schering Pharma AG, Berlin, Germany).

Incubation of ferritin in the presence of GBCA
The same buffer approximating physiological conditions (50 mM HEPES, pH 7.4, 0.9% NaCl) was used for all incubations of ferritin (ferritin from equine spleen, Type I, Sigma-Aldrich, St. Louis, MO; 4.2 mg/ml [5 μM]) with gadolinium-chelates (24 h at 37 °C). Samples for HAADF-STEM imaging and SQUID measurements were prepared with 14.3 mM **1**; all other samples were prepared with 1.43 mM GBCA. For control apoferritin samples (apoferritin from equine spleen, Sigma-Aldrich, St. Louis, MO), the same procedure and protein concentration were used. Another control apoferritin sample was prepared under the same conditions but with an extreme **1** concentration of 143 mM. Incubations were always followed by repeated dialysis (cut off 12–14 kDa, 4 days, four buffer changes, individual dilution factor 300, total dilution 10^8-fold). The results are summarized in Table S1.
A part of the sample of equine ferritin incubated with **1** was purified by gel permeation chromatography (column Superose 6 10/300 GL, flow rate 0.5 ml/min, 50 mM HEPES, pH 7.4, 0.9% NaCl, injection 100 μl) instead of by dialysis. The ferritin fraction was collected. A control sample of **1** (without ferritin) was injected on the same column. The fraction corresponding to the elution of ferritin was collected. Both samples were analyzed in the same manner as the dialysed samples. The results are summarized in Table S2.
An additional set of samples comparing equine and human ferritin (native human ferritin from liver, Bio-Rad Laboratories, 4.2 mg/ml [5 μM]) was incubated with 1.43 mM **1** in the above described HEPES buffer and in human serum (from human male AB plasma, USA origin, Sigma-Aldrich) and purified by repeated dialysis. The results are summarized in Table S3.
Gadolinium and iron concentrations were measured with inductively coupled plasma mass spectrometry (ICP MS 7700, Agilent Technologies). Each reaction was analyzed in triplicate. In control experiments, solutions of GBCA were dialyzed in the absence of ferritin using the same procedure. In these cases, Gd concentrations were negligible (at least 2–3 orders of magnitude lower compared to the incubated ferritin fractions). The protein concentrations were determined by amino acid analysis using ion-exchange chromatography on a Biochrom-30 amino acid analyser with ninhydrin detection.

HAADF-STEM imaging
TEM specimens were made by drop-casting samples directly onto holey carbon film-coated TEM grids and dried at room temperature for 15 min. The specimens were analyzed with a JEOL ARM scanning transmission electron microscope operated at 200kV. Imaging was carried out in aberration-corrected high-angle annular dark field (HAADF) STEM mode, with a probe size of approximately 0.08 nm and a collection angle of 67-250 mrad. The dwell time of the scanning beam was kept deliberately low, and high spot size was used to reduce the total electron dose on the specimen. These conditions allow sufficient spatial resolution for imaging a single Gd atom, and the contrast mechanism is sensitive to elements with high atomic number (so-called Z-contrast imaging).

SQUID magnetometry
Magnetic measurements were carried out with an MPMS7XL SQUID magnetometer (Quantum Design, USA). Samples were measured in both liquid and solid form to evaluate the influence of freeze-drying on the magnetic performance. In a typical experiment, a liquid sample containing approximately 1 mg of the magnetically active substance was placed in a homemade sample holder with low background signal and promptly loaded into the chamber, which was equilibrated at 250 K. The chamber was

repeatedly evacuated and flushed with He gas to avoid contamination with air, which has a strong paramagnetic signal below ~50 K.
The sample was cooled down to 2 K in zero magnetic field (MF). Then, a MF (0.01 T, 0.1T and 1 T) was applied and the temperature dependence of magnetization (zero field-cooled, ZFC) was recorded during a 0.5 K/min temperature sweep over the interval 2 – 100 K with a step of 0.5 K. The sample was then cooled back to 2 K, and the temperature dependence of magnetization (field-cooled, FC) was recorded in the interval 2 – 20 K using the same sweep and sampling rates. Next, a full hysteresis loop was recorded at 2 K in a MF up to ± 7 T. A set of magnetization isotherms above the blocking temperature (T_B) up to 7 T was measured at 50, 100, and 200 K. Solid samples were placed in a gelatine capsule, loaded into a sample holder and measured following the same protocol as for liquid samples.
Before fitting procedures were applied, the experimental data were checked for invariance in the (super)paramagnetic regime, corrected for demagnetization factor and background signal (liquid samples), and normalized. All fittings of corrected experimental data were carried out using homemade routines in Octave software.[1]
The basic parameters of the single-domain (maghemite) component were determined from the ZFC curve. The mean blocking temperature of the maghemite phase (T_B^m) was derived using the general formula:

$$M_{ZFC}(T) \propto A + B\left[\frac{25}{t}\int_0^t t_B f(t_B)dt_B + \int_t^\infty f(t_B)dt_B\right], \qquad (1)$$

where A is a correction to the random orientation of the freezing moments, resulting in a random nonzero value of the low temperature magnetization, and B is a fraction-scaling constant, which is refined together with T_B^m and σ, as the exact values of M_S and K_{eff} are usually not known. The effective anisotropy constant was estimated using the term:

$$K_{eff} = k_B T_B^m / V_{mag}, \qquad (2)$$

where k_B is the Boltzmann constant and V_{mag} is the magnetically active volume of the particle related to the so-called magnetic size (diameter, d_{mag}), given by the expression:

$$d_{mag} = \sqrt[3]{\frac{6\mu_1 a^3}{\mu_{uc}\pi}} = 2\sqrt[3]{\frac{3k_B T_B^m}{4\pi K_{eff}}} = \sqrt[3]{\frac{6V_{mag}}{\pi}}, \qquad (3)$$

where a and μ_{uc} are the lattice parameter and the magnetic moment of the unit cell of the maghemite phase, respectively.[2]
The magnetization isotherms above T_B^m were subjected to analysis by the standard two-component model[3] and updated two-component model (further discussed in Additional Results and Discussion):

i) The standard two-component model (abbreviated according to 'two-Langevin' as 2L) assumes a sum of two Langevin functions, L_1 and L_2 (relevant only for the *classical* spin limit of large J), and a linear term:

$$M_{2L} = CL_1(x_1) + DL_2(x_2) + \chi_c H. \qquad (4)$$

The first and second terms account for maghemite and ferrihydrite, respectively; x_1 and x_2 are the arguments of the corresponding Langevin functions scaling with the magnetic moment, μ as: $x = \mu_0 H g \mu / k_B T$ (μ_0 – permeability of vacuum, g – Lande g-factor); and C, D are the scaling factors proportional to the saturation magnetization of each magnetic phase. The last term accounts for the dominant linear contribution present in the experimental data; however, the physical relevance of this term is not clearly justified.[3]

ii) The modified two-component model (abbreviated according to 'Brillouin-Langevin' as BL) considers the intrinsic magnetic structure of the ferrihydrite phase in ferritin, and thus assumes a sum of Langevin and Brillouin functions:

$$M_{LB} = M_1(x_1) + FBj(x_2). \qquad (5)$$

The first and second terms account for maghemite and ferrihydrite, respectively; x_1 and x_2 are the arguments of the corresponding functions defined above; and E, F are the scaling factors proportional to the saturation magnetization of each magnetic phase. The magnetization of the maghemite fraction (M_1) was fitted as a weighted sum of the Langevin functions:

$$M_1(H,T) = \int_0^\infty \mu L\left(\frac{\mu H}{k_B T}\right) f(\mu) d\mu + \chi_{linear} H, \qquad (6)$$

where $f(\mu)$ corresponds to the unimodal log-normal distribution of the magnetic moments, and μ is expressed as:

$$f(\mu) = \frac{1}{\sqrt{2\pi}\mu\sigma} exp\left(- \frac{ln^2\left(\frac{\mu}{\mu_m}\right)}{2\sigma^2} \right), \mu_{md} = \mu_m exp\frac{\sigma^2}{2} \quad (7)$$

where σ is the distribution width, and μ_m and μ_{md} are the median and mean magnetic moment, respectively. The second term in eq. (6) corresponds to a correction that originates from the paramagnetic components of the sample (usually the disordered parts of the particle) or diamagnetic impurities and usually accounts for less than 5% of the total magnetization. This term has a physical relevance and represents only a minor correction in comparison to a similar term in eq. (4).

iii) Finally, correction to the Gd^{3+} content was modelled by a single Brillouin function assuming a quantum spin of $S = 7/2$:

$$M_{2L}^{Gd} = XM_{LB} + G_{2L}Bj(x_3) \quad (8a)$$

and

$$M_{LB}^{Gd} = YM_{LB} + G_{BL}Bj(x_3). \quad (8b)$$

X, Y are the scaling factors proportional to the ferritin magnetization in the 2L and BL models, and G_{2L} and G_{BL} describe the Gd^{3+} content for each model.

Electronic paramagnetic resonance (EPR)

EPR spectra were recorded on an EMX^{plus}-10/12 CW (continuous wave) EPR spectrometer (Bruker, Germany) equipped with a Premium-X-band microwave bridge. Each sample was prepared in a quartz suprasil EPR tube (O.D. 4 mm, Wilmad Lab-glass, US) and immersed in liquid N_2 within a Dewar finger vessel, which was then inserted into the standard rectangular EPR cavity (ER-4102003-ST, Bruker, Germany). A solution of **1** was diluted with HEPES buffer to a final concentration of 50 μM; the Gd^{3+}-ferritin nanoparticles or ferritin were added to final concentrations of 1.00 mg/ml or 0.305 mg/ml, respectively. After conditioning in the Dewar finger vessel (2-4 min) directly in the EPR cavity, spectra were recorded using the following instrumental parameters: microwave frequency = 9.5397 GHz, central field = 120 mT, sweep width = 180 mT, receiver gain = 1.10^4, modulation amplitude = 0.5 mT, modulation frequency = 100 kHz, power = 20 mW, resolution = 3601 points, conversion time = 24.0 ms, and time constant = 5.1 ms. Each spectrum was recorded as the accumulation of 6-12 sweeps to increase the signal-to-noise ratio.

Calculation of free Gd^{3+} concentrations

To evaluate the thermodynamic stability of the chelates, a direct comparison of stability constant values is rather misleading, as complexation is a concurrent process to donor atom protonation, and ligand basicity must therefore be taken into account. To objectivize metal binding by various ligands, we calculated the concentration of free Gd^{3+} (aqua)ion (pGd = – log[Gd^{3+}]) in solutions of given chelates under our incubation conditions.

The protonation constants of the ligands and stability constants of their Gd^{3+} chelates were taken from previously published data (for references, see Table S4). The species distributions were calculated using the OPIUM program package.[4] When protonation and stability constants came from different published sources, the calculation was performed using constants obtained employing the same experimental conditions (temperature, ionic strength). Calculations were performed for pH = 7.4 and c(GBCA) = 1.43 mM. The pGd = –log[Gd^{3+}] values were calculated for simple Gd–ligand systems without taking the influence of other metal ions into account, as no other (bio)metal ions were present in the ferritin/GBCA incubation experiments. The value of pK_w used was 13.78, and the stability constant of Gd-hydroxidocomplex $[Gd(OH)]^{2+}$ was $\log\beta_{-101} = -8.40$.[5]

A compilation of all literature data and the corresponding pGd values are outlined in Table S4. The literature data show certain variances, as different conditions were used in thermodynamic studies. Only the most relevant (NaCl used for adjustment of ionic strength) and/or the most frequent (when contradictory protonation and stability constants were reported) results were chosen for the representative comparison shown in Table S5. Nevertheless, all of the various calculated pGd values (Table S4) for each chelate support the discussion of thermodynamic versus kinetic stability presented in the main text.

Determination of ^1H relaxation times in the presence of Gd^{3+}-ferritin nanoparticles

T_1 and T_2 relaxation times for ^1H in an aqueous solution of Gd^{3+}-ferritin nanoparticles were measured on a Bruker AVANCE III spectrometer (Bruker, Germany) equipped with a cryoprobe operating at 500.0 MHz for ^1H. Inversion recovery (180° pulse–delay–90° pulse) was used for T_1 measurements with a relaxation delay ranging from 0.1 s to 10 s. Protein solutions (500 μl with a concentration of 1.00 mg/ml in HEPES buffer were measured with 8 scans and a 15-s pre-scan delay (d1). The inversion recovery experiment was repeated nine times, and the resulting T_1 values were averaged. T_2 relaxation times were measured with a Carr-Purcell-Meiboom-Gill (CPMG) sequence (90° pulse–delay–180° pulse–delay) with 1–10 repeats of the delay–pulse-delay segment and a fixed inter-echo time of 10 ms. The experiment was repeated nine times, and the resulting T_2 values were averaged. Protein

solutions (500 μl) with a concentration of 1.00 mg/ml in partly deuterated HEPES buffer (D_2O:H_2O volume ratio 9:1) were measured with 8 scans and a 10-s d1. T_2^* relaxation times were estimated from the lineshape of the water signal; the experimental signal was fitted with a simulated Lorentzian signal, and T_2^* was calculated according to the equation $T_2^* = 1/FWHM*\pi$, where FWHM is full width at half maximum of the simulated signal.

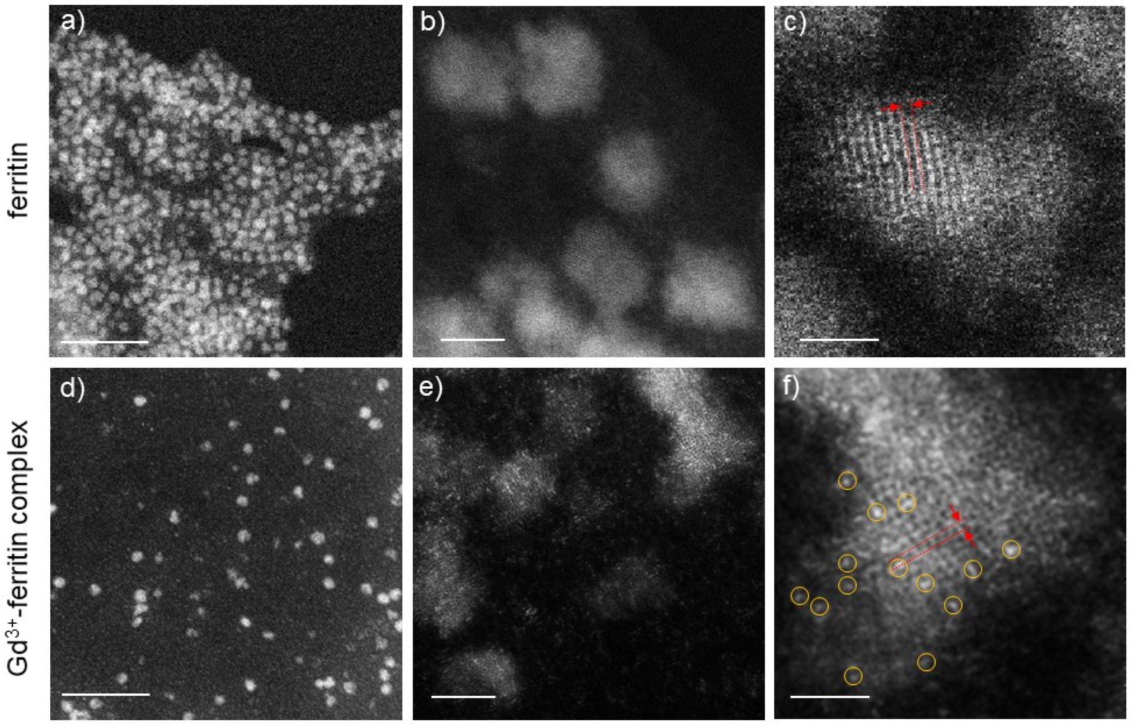

Figure S1. Additional HAADF-STEM images of a-c) ferritin and d-f) Gd^{3+}-ferritin. The iron oxyhydroxide cores are imaged in a) and d) (scale bar: 50 nm) and b) and e) (scale bar: 5 nm) as bright contrast. In comparison with Gd^{3+}-ferritin, images of ferritin cores do not have bright spots. High-resolution images c) and f) show lattice fringes and the atomic columns of ferrihydrite (scale bar: 2 nm). The arrows indicate 2.6 Å, the {110} spacing of ferrihydrite.[6] The yellow circles in f) mark sites with particularly high intensities, indicating the presence of Gd atoms in Gd^{3+}-ferritin. Note the intensities in c) for ferritin do not show the presence of very bright spots. Due to the polycrystallinity of the cores,[7] the atomic columns in some parts of the cores are not resolved**.**

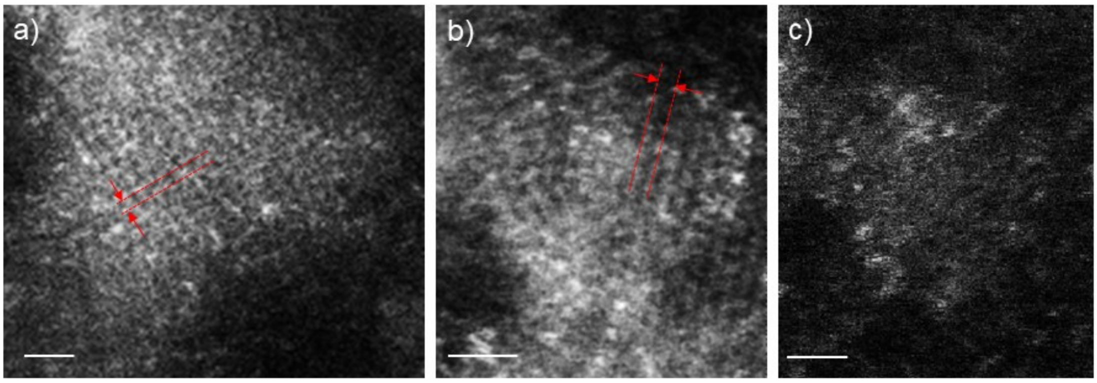

Figure S2: Additional HAADF-STEM images of Gd^{3+}-ferritin nanoparticles. All the particles have particularly bright spots within the cores, confirming the consistency of the presence of Gd ions within ferritin cores. a-b) The arrows between the red dotted lines indicate the {110} spacing of ferrihydrite.[6] c) A core out of the zone axis (therefore absence of the lattice lines) also shows the presence of Gd ions. Scale bar: 1 nm.

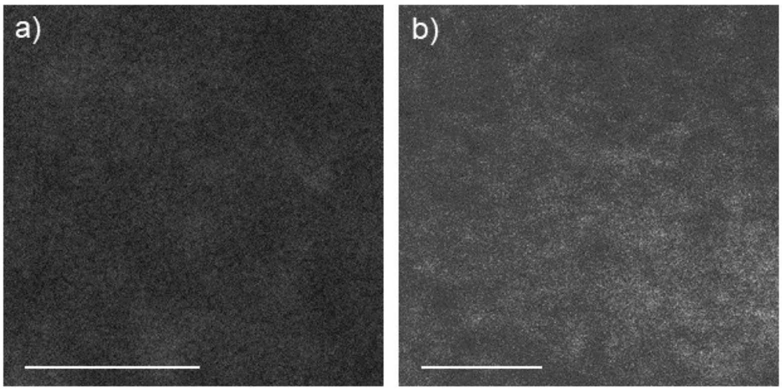

Figure S3: HAADF-STEM images of apoferritin incubated with **1**. As apoferritin is composed of only light scattering elements, it is not expected to show any contrast if Gd or Fe atoms are present. a) Lower magnification image with uniform contrast (scale bar: 50 nm); b) Higher magnification image. There is no contrast arising from higher Z elements (such as Gd or Fe), suggesting that apoferritin does not contain any Gd (scale bar: 5 nm).

Table S1. Concentrations of protein, Gd and Fe in retentates analyzed after incubations and repeated dialysis. Note that the concentration of Gd in the control sample is ~6 orders of magnitude lower than original 1.43 mM concentration of **1**, reflecting sufficient removal of excess unreacted **1** by dialysis. Binding of Gd^{3+} (or possibly also undissociated **1**) to apoferritin is negligible compared to the amount of Gd^{3+} bound to ferritin. To assess whether this binding is concentration-dependent, we also tested an extreme, 100x higher concentration of **1** (143 mM). The *n*(Gd)/*n*(protein) ratio was not significantly different from that of incubations with 1.43 mM **1**, suggesting binding of Gd^{3+} to the protein is non-specific and negligible compared to the binding to the ferrihydrite lattice. Molecular weights used for calculations—ferritin: 830,000 g/mol, apoferritin: 450,000 g/mol. The errors for concentration are expressed as standard deviations from three measurements. The errors for ratios were calculated as combined standard uncertainties using error propagation rules for division.

**Sample**	***c*(protein)** (μM)	***c*(Gd)** (nM)	***c*(Fe)** (μM)
Ferritin	4.21 ± 0.05	17 ± 3	3 043 ± 73
Ferritin + 1.43 mM **1** (sample 1)	2.41 ± 0.20	16 900 ± 90	1 694 ± 35
Ferritin + 1.43 mM **1** (sample 2)	2.35 ± 0.32	17 600 ± 300	1 892 ± 48
Apoferritin + 1.43 mM **1** (sample 1)	3.01 ± 0.28	443 ± 18	27.2 ± 2.8
Apoferritin + 1.43 mM **1** (sample 2)	3.33 ± 0.17	496 ± 100	25.9 ± 4.7
Apoferritin + 143 mM **1**	3.29 ± 0.32	530 ± 10	28.0 ± 1.2
Control (1.43 mM **1**)	—	8 ± 0	24.4 ± 1.8

**Sample**	***n*(Gd)/*n*(protein)**	***n*(Fe)/*n*(protein)**	***n*(Fe)/*n*(Gd)**
Ferritin	0.004 ± 0.001	724 ± 20	175 000 ± 28 000
Ferritin + 1.43 mM **1** (sample 1)	7.012 ± 0.587	704 ± 61	100.3 ± 2.1
Ferritin + 1.43 mM **1** (sample 2)	7.486 ± 1.012	805 ± 110	108 ± 3.2
Apoferritin + 1.43 mM **1** (sample 1)	0.148 ± 0.015	9.0 ± 1.3	61 ± 6.8
Apoferritin + 1.43 mM **1** (sample 2)	0.149 ± 0.031	7.8 ± 1.5	52 ± 14
Apoferritin + 143 mM **1**	0.161 ± 0.160	8.5 ± 0.9	53 ± 2.5
Control (1.43 mM **1**)	—	—	3 118 ± 231

Table S2. Comparison of two alternative methods considered for purification of Gd^{3+}-ferritin: dialysis and GPC. Concentrations of Gd and Fe were analyzed after incubations in: 1) retentates from repeated dialysis, 2) ferritin fraction eluted from GPC column (see Experimental procedures for details). Both methods provided identical Fe/Gd ratios in the Gd^{3+}-ferritin. Note that the concentration of Gd in the control sample is 5 orders of magnitude lower than the original 1.43 mM concentration of **1**, reflecting sufficient removal of excess unreacted **1** by both methods. The identical purification efficacy of both methods led us to use dialysis in all other experiments, due to the 36-fold higher concentration of ferritin (and thus concentrations of Fe and Gd) in the samples purified by dialysis and the possibility of straightforward scalability of Gd^{3+}-ferritin preparation.
The errors for concentration are expressed as standard deviations from three measurements. The errors for ratios were calculated as combined standard uncertainties using error propagation rules for division.

**Sample**	***c*(Gd)** (nM)	***c*(Fe)** (µM)	***n*(Fe)/*n*(Gd)**
1) Dialysis			
Ferritin + 1.43 mM **1**	21 100 ± 410	3 582 ± 76	170 ± 5.0
Control (1.43 mM **1**)	7.0 ± 0.31	8.0 ± 0.16	1 135 ± 55
2) GPC			
Ferritin + 1.43 mM **1**	587 ± 12	98.5 ± 1.16	168 ± 4.0
Control (1.43 mM **1**)	2.0 ± 0.12	1.45 ± 0.05	740 ± 52

Table S3. Concentrations of protein, Gd and Fe in retentates analyzed after incubations of equine and human ferritins with **1**. HEPES buffer and human serum were used as two different reaction environments. After incubation, the samples were purified by repeated dialysis. The presence of the serum proteins led to a decrease in total loads *n*(Gd)/*n*(protein) by a factor of ~3. Molecular weights used for calculations—ferritin: 830,000 g/mol, apoferritin: 450,000 g/mol. The errors for concentration are expressed as standard deviations from three measurements. The errors for ratios were calculated as combined standard uncertainties using error propagation rules for division.

**Sample**	***c*(protein)** (µM)	***c*(Gd)** (nM)	***c*(Fe)** (µM)
50 mM HEPES, pH 7.4, 0.9% NaCl, 37 °C			
Equine ferritin + 1.43 mM **1**	1.86 ± 0.23	21 100 ± 410	3 582 ± 76
Human ferritin + 1.43 mM **1**	1.62 ± 0.13	13 900 ± 120	1 843 ± 16
Human serum			
Equine ferritin + 1.43 mM **1**	1.98 ± 0.25*	7 340 ± 105	3 812 ± 91
Human ferritin + 1.43 mM **1**	1.80 ± 0.15*	4 800 ± 75	2 048 ± 50

**Sample**	***n*(Gd)/*n*(protein)**	***n*(Fe)/*n*(protein)**	***n*(Fe)/*n*(Gd)**
50 mM HEPES, pH 7.4, 0.9% NaCl, 37 °C			
Equine ferritin + 1.43 mM **1**	11.3 ± 1.4	1 925 ± 243	170 ± 5
Human ferritin + 1.43 mM **1**	8.6 ± 0.7	1 139 ± 94	133 ± 2
Human serum			
Equine ferritin + 1.43 mM **1**	3.7 ± 0.5	—*	519 ± 14
Human ferritin + 1.43 mM **1**	2.7 ± 0.2	—*	427 ± 12

* The concentrations of ferritin in serum were determined based on iron contents after subtraction of iron background concentration in serum. Because the quantification of ferritin concentration by amino acid analysis is possible in samples containing a pure protein only, the values *n*(Fe)/*n*(protein) in samples containing serum cannot be estimated using amino acid analysis.

Table S4. Protonation constants (β_h [a]) of ligands used in GBCAs, stability constants (β_{hlm} [b]) of corresponding GBCAs (Gd^{3+} chelates) and calculated pGd values under the following conditions: c(Gd^{3+}) = c(Ligand) = 1.43 mM, pH = 7.4. Various sets of stability constants based on previously published data were used for each chelate.

GBCA	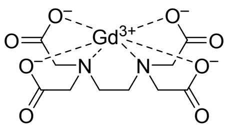 **7** (Gd-EDTA)		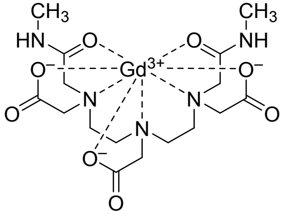 **1** (Omniscan)		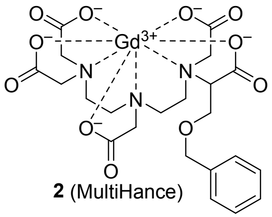 **2** (MultiHance)				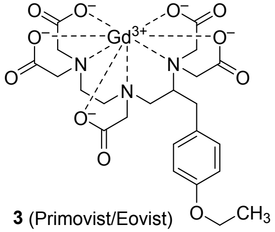 **3** (Primovist/Eovist)
Ionic strength	0.1	0.1 NMe$_4$Cl	0.1 NaCl	0.15 NaCl	0.1 NMe$_4$NO$_3$	0.15 NaCl	0.1 KCl[c]	0.15 NaCl	0.1 NMe$_4$Cl
Reference	5	8	9	10	11	11	12	10	11
logβ_1	10.19	10.11	9.37	8.98	10.86	10.76	10.71	9.85	11.52
logβ_2	16.32	16.30	13.75	13.34	19.15	18.93	18.98	17.76	20.35
logβ_3	19.01	19.17	17.06	16.55	23.49	23.24	23.33	22.05	24.80
logβ_4	21.01	21.43	18.49	18.05	26.27	25.93	26.16	24.88	27.75
logβ_5	22.51	–	–	–	28.50	28.11	28.23	26.84	30.20
logβ_6	22.51	–	–	–	–	–	–	–	–
logβ_7	–	–	–	–	–	–	–	–	–
logβ_{011}	17.35	17.7	16.85	16.64	22.58	22.61	22.59	21.91	22.76
logβ_{111}	18.65	–	–	–	24.33	–	–	23.56	–
logβ_{-111}	–	–	–	–	–	–	–	–	–
**pGd**	**8.711**	**8.925**	**8.881**	**8.967**	**10.532**	**10.649**	**10.620**	**10.859**	**10.040**

GBCA	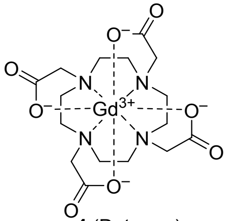 **4** (Dotarem)						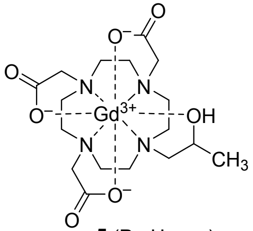 **5** (ProHance)			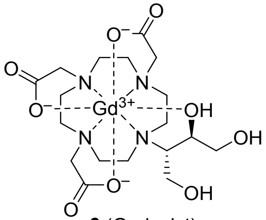 **6** (Gadovist)		
Ionic strength	0.1 K(+)	0.1 NaCl	0.1 KCl	0.1 NMe$_4$NO$_3$	0.1 NMe$_4$Cl	0.1 NMe$_4$Cl	0.1 NMe$_4$Cl	0.1 NMe$_4$Cl	0.1 NMe$_4$Cl	0.1 NaCl	0.1 NMe$_4$Cl	0.1 KCl
Reference	5	8	13,14	15	8	16	8	17	16	18	18	18
logβ_1	11.20	9.37	11.14	12.12	11.73	11.74	11.96	10.89	11.17	9.46	11.75	11.46
logβ_2	20.93	18.51	20.83	21.79	21.13	21.5	21.39	20.68	20.50	18.82	20.98	20.75
logβ_3	25.37	23.14	25.68	26.34	25.63	26.18	25.69	24.97	25.49	22.99	25.11	24.89
logβ_4	29.71	27.05	29.63	30.48	29.82	30.29	28.95	–	29.29	26.01	28.08	27.85
logβ_5	32.06	–	–	–	–	32.66	–	–	32.13	–	–	–
logβ_6	33.76	–	–	–	–	–	–	–	–	–	–	–
logβ_{011}	24.0	24.6	24.0	27.0	25.3	24.67	23.8	22.8	24.5	18.70	20.8	21.8
logβ_{111}	26.3	–	26.3	–	–	–	–	–	–	19.82	–	–
logβ_{-111}	–	–	–	–	–	–	–	–	–	9.22	–	–
**pGd**	**10.377**	**11.884**	**10.426**	**11.447**	**10.926**	**10.427**	**10.046**	**9.902**	**10.840**	**8.782**	**8.750**	**9.365**

[a] $\beta_h = [H_hL] / ([H]^h \cdot [L])$; [b] $\beta_{hlm} = [H_hL_lM_m] / ([H]^h \cdot [L]^l \cdot [M]^m)$; [c] 20 °C

Table S5. Comparison of concentrations of free Gd^{3+} ions (expressed as pGd) with chelate charges and the observed Gd^{3+}/ferritin loadings in a physiological environment. Representative pGd values were selected from Table S4. Compare with Figure 3 in the main text.

Chelate	pGd	Gd^{3+}/ferritin	Chelate charge
**7**	8.7	43	1–
**1**	9.0	7.2	0
**2**	10.6	0.89	2–
**6**	8.8	0.31	0
**3**	10.0	0.10	2–
**5**	9.9	0.010	0
**4**	10.4	0.0026	1–

Additional Results and Discussion

SQUID magnetometry

Modelling the magnetic properties of commercial equine spleen ferritin is often based on the assumption of a single-phase core of ferrihydrite ($5Fe_2O_3 \cdot 9H_2O$), a non-interacting antiferromagnet (AF) with a magnetic ordering temperature, or Neel temperature (T_N), of ~12–20 K. However, low-temperature magnetic measurements have indicated that the ferritin cores contain at least two magnetic phases, and thus improved models include a sum of two or three components expressed as a Langevin function (model function for magnetization of a non-interacting spin in the *classical* limit).[3,19] The two-phase model reported by Brem and coworkers[3] consists of a phase with a high coercivity that does not undergo saturation and a second phase with a low coercivity and low saturation field. The high-coercivity phase is compatible with antiferromagnetic ferrihydrite, while the low-coercivity phase corresponds to the iron sesquioxide phase. The antiferromagnetic and ferrimagnetic fingerprints also have been identified in the Mössbauer spectra of ferrihydrite, and the two components are associated with varying amounts of ferric ions in individual ferritin particles.[20] Additionally, the magnetic properties of ferrihydrite can change dramatically with particle size,[21] most likely due to structural and spin disorder.[2]

One controversial point is the use of a Langevin function to describe an AF phase in a paramagnetic regime (we analyzed a magnetization isotherm at 50 K). While the use of a Langevin function is physically relevant for description of a superparamagnetic state (which explicitly means a state of a single-domain part of a ferromagnet or a ferrimagnet above the T_B), an AF cannot enter the superparamagnetic regime due to lack of magnetic domains, and thus competition between the domain wall energy and magneto-static stabilization energy.[22,23] Therefore, it follows a standard Curie (or Curie-Weiss) law above the T_N, and a Brillouin function derived for a *quantum* spin should be used to describe magnetization. In other words, a Langevin function should not be used to describe magnetization of ferrihydrite. Considering this fundamental conflict, we formulated the BL model [eq. (5)] by replacing the Langevin function in the 2L model [eq. (4)] with a Brillouin function accounting for AF in the paramagnetic state. The relevance of using the Brillouin function to describe the paramagnetic phase of ferritin has been reported.[24] To account for the particle size distribution of maghemite, the Langevin function in the BL model was replaced with a magnetic moment-weighted sum (integral) of Langevin functions.

Substitution of the Langevin function with the Brillouin function to describe the ferrihydrite phase in the paramagnetic region has physical relevance and gives the best fit without need for arbitrary corrections. The linear correction in eq. (5) represents about 90-98% of the total magnetization, which underscores the relevance of our BL model. The shape of the Brillouin function (shown in Figure S4) follows a perfectly linear trend at the given temperature and magnetic field range. This points to the match with the arbitrary linear term used in the 2L model [eq. (4)]. It is likely an independent proof for our BL model, which suggests that the 2L model could be in many cases simplified to the sum of a Langevin function (or weighted distribution) and a linear term. We tested both models to better compare our results with published data. The results of the fits are summarized in Table S6. The BL model yields slightly better fit quality, but both models describe the experimental data satisfactorily.

Our experimental data obtained for pure equine spleen ferritin (Figure S4) are in good agreement with previous results.[3,19] The magnetization data (Figure S4) suggest that the ferritin used in this study consists of very small single-domain particles (maghemite) and a paramagnetic phase (disordered ferrihydrite). This is supported by a single bifurcation point at the ZFC and FC curves related to the blocking of the single-domain (maghemite) nanoparticles, and lack of additional anomaly or bifurcation due to AF (or more complex) ordering of ferrihydrite at higher temperatures. The proposed phase composition is corroborated by the character of the magnetization isotherm (hysteresis loop) at 2 K, which does not tend to saturate and shows a moderate coercivity of approximately 0.1 T. The hysteresis curves of the Gd^{3+}-containing samples show a general suppression of necking and appearance of a clear paramagnetic Brillouin-like contribution due to the Gd^{3+} phase, which yielded estimates of Gd^{3+} content per one particle of Gd^{3+}-ferritin nanoparticle (Table S6). The difference in Gd

content is not statistically significant for the correction applied to the 2L and BL models, as they both satisfactorily describe the shape magnetization isotherm.

As discussed in the main text, the ZFC-FC curves of the Gd^{3+}-containing samples show the clear signature of an additional paramagnetic term due to Gd^{3+} ions in the ferritin particle shell. The values of μ_{2L1}, μ_{2L2} and μ_{BL} are almost constant upon Gd^{3+} insertion (Table S6), suggesting Gd^{3+} incorporation on the surface of the ferritin particle. To put this in context with real particle dimensions, the calculated magnetic size (d_{mag}) was identical within experimental error for all samples (Table S6).

Considering a typical value for the effective anisotropy constant for maghemite in nanoparticles with diameter below 10 nm (K_{eff} = 5×10^5 J/m^3), the d_{mag} of the maghemite single-domain in ferritin can be estimated using eq. (3), yielding a value of about 7 nm, which is somewhat consistent with previous studies,[3,19] but two times larger than the value yielded by magnetization analysis. This value is related to the magnetically aligned fraction, and thus must be lower than the diameters obtained from TEM or XRD analysis,[2] while previous studies refer to the physical size of the particles. In light of analysis of the magnetization isotherms, the moderate increase in T_B^m likely occurs due to the increase in K_{eff} after Gd^{3+} incorporation into the ferritin core. The relative change can be estimated using the relation: K_{eff} (Gd^{3+}) / K_{eff} = T_B^m / T_B^m(Gd^{3+}), which yields an approximately 8% increase in the effective anisotropy. A similar effect has been observed for La-substituted ferrite nanoparticles.[25]

Finally, we addressed the possible phase accommodation in the ferritin core, schematically shown in Figure S4. The entire core has a physical diameter of about 8 nm. The clear signature of single-domain particles of the maghemite phase suggest that maghemite must form particles of at least 3 nm (lower limit given by the d_{mag}). Considering a typical level of structural disorder in small maghemite particles,[3] the physical diameter can reach a value of about 4 nm. This indicates that the central part of the core is most likely formed from a single maghemite nanoparticle and the disordered ferrihydrite phase fills the outer part within the ferritin cavity. This scenario is also corroborated by experiments carried out on Gd^{3+}-ferritin nanoparticles. The d_{mag} does not vary upon Gd^{3+} insertion, while a moderate change in effective anisotropy occurs, which is related to the reforming of the outer disordered part of the magnetic core.

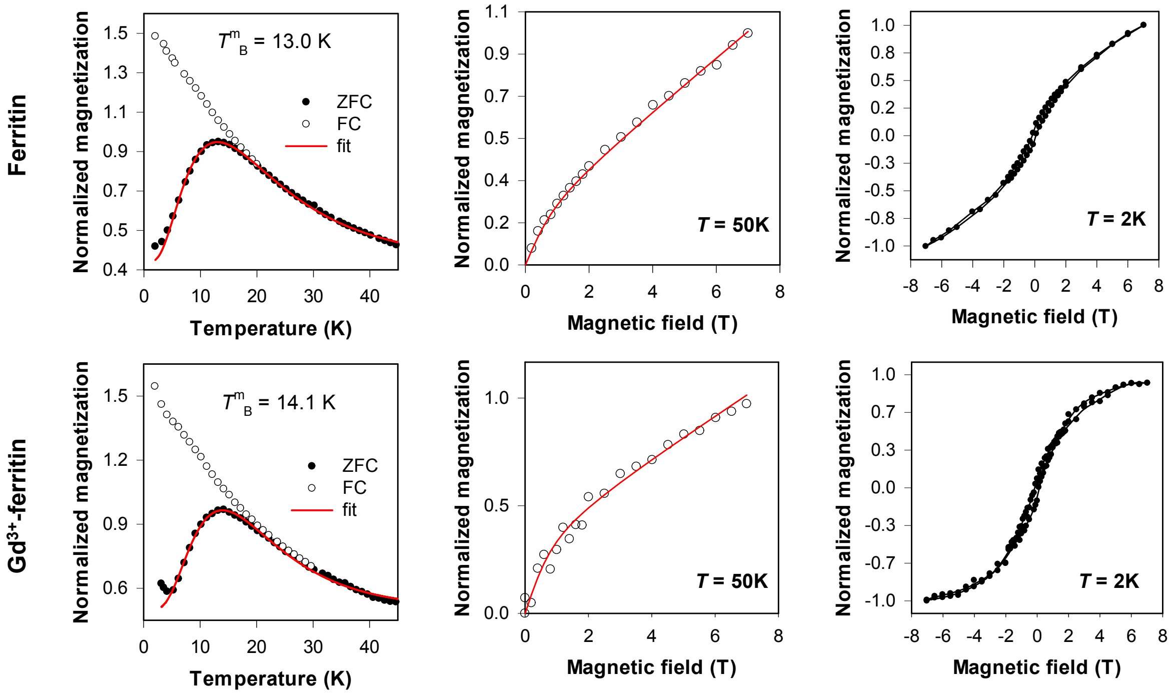

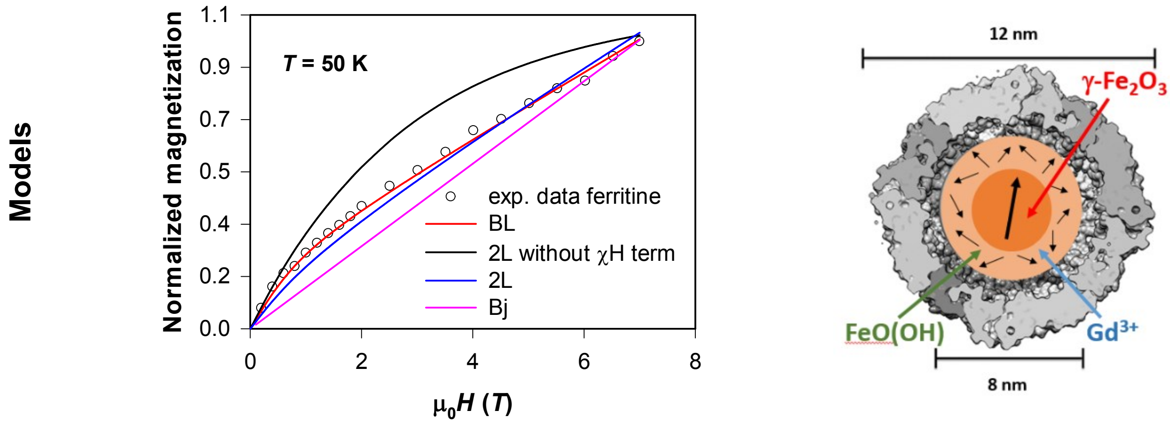

Figure S4. Magnetic measurements for ferritin (first row) and Gd^{3+}-ferritin nanoparticle (prepared upon incubation with 14.3 mM **1**; second row). The red lines correspond to fits according to eq. (1) and (8b), respectively. The last row shows (left) a comparison of the models applied to ferritin at 50 K: 2L - eq. (4), BL - eq. (5), 2L - eq. (4) without the arbitrary χH correction, and Brillouin function (Bj) for S = 5/2; the data are normalized to magnetization at 7 T. A sketch of the rationalized magnetic phase distribution in ferritin is shown on the right.

Table S6. Summary of selected magnetic parameters of ferritin and Gd^{3+}-ferritin nanoparticles obtained from analysis of SQUID magnetometry: mean blocking temperature (T_B^m); contributions of ferritin according to 2L (μ_{2L1} and μ_{2L2}) and BL [μ_{LB} – corresponds to μ_m in eq. (7)] models; estimate of the number of Gd^{3+} /ferritin according to eq. (8b); and magnetic size of the maghemite phase (d_{mag}) calculated from μ_{LB} using eq. (3).

	T_B^m [K]	$\mu_{2L1}[\mu_B]$	$\mu_{2L2}\,[\mu_B]$	$\mu_{LB}\,[\mu_B]$	σ	Gd^{3+} /ferritin	d_{mag} [nm]
Ferritin	13.0±0.2	340±34	690±69	630±63	0.2	0	3.0±0.3
Ferritin incubated with 1.43 mM **1**	13.9±0.2	352±52	721±72	642±42	0.2	1±5	3.0±0.3
Ferritin incubated with 14.3 mM **1**	14.1±0.2	339±34	683±68	618±62	0.2	9±2	3.0±0.3

EPR measurements

Ferritin, free GBCA **1** and Gd^{3+}-ferritin nanoparticles were investigated using EPR in high and low field spectral regions. In ***higher fields*** (g-factor region 3.59–1.39, corresponding to 190 to 490 mT, respectively), ferritin and Gd^{3+}-ferritin displayed characteristic spectral lines for Fe^{3+}. EPR spectra of pure **1** resembled the so-called "U-type" ("U" stands for ubiquity; data not shown).[26–30] However, the EPR spectral lines for ferritin and Gd^{3+}-ferritin within the high-field range were too broad and possessed a lower intensity than those at low fields. The high-field region was not suitable for simultaneous identification of Gd^{3+} and Fe^{3+} because their signals overlap.

In contrast, the ***low-field spectral parts*** (g-factor region 9.74–3.59) of ferritin, free GBCA **1** and Gd^{3+}-ferritin provided more detailed information (Figure S5). The ferritin sample exhibited one Fe^{3+} EPR signal at $g = 4.27$ with a linewidth of $\Delta B_{pp} = 3.25\ mT$ (measured as peak-to-peak distance). This signal is characteristic for high spin ($S = 5/2$) ferric ions surrounded by ligand atoms within the ferritin structure and possessing a low rhombic symmetry.[31–33] The Gd^{3+} signal was found at $g = 5.87$ ($\Delta B_{pp} = 7.43\ mT$) and $g = 5.82$ ($\Delta B_{pp} \approx 8.7\ mT$), corresponding to **1** and Gd^{3+}-ferritin, respectively. The ground state of Gd^{3+}, corresponding to $^8S_{7/2}$, possesses a valence $4f^7$ electronic configuration with the overall electron spin quantum number $S = 7/2$.[26] The signal at $g \approx 6$ (in our case $g = 5.87$ and $g = 5.82$) corresponded to the $7/2 \leftrightarrow 5/2$ spin transition,[27,34] and its position depended on the zero-field splitting D, which in our case and according to *Mazur et. al.*,[28] falls into the region of $D = (0.045 - 0.055)\ cm^{-1}$. These characteristic Gd^{3+} EPR spectra were consistent with Gd-coordination number >6, and these centers had no specific defined site symmetries.[26,27,29,30]

The Gd^{3+} line at $g = 5.82$ for Gd^{3+}-ferritin was rather broad and displayed a lower signal-to-noise ratio (most likely due to a lower Gd^{3+} concentration and faster electron spin relaxation within ferritin). Therefore, it was not possible to obtain more accurate characteristics. For this reason, our spectral interpretation also omitted the hyperfine coupling by magnetically active nuclei such as ^{57}Fe, ^{155}Gd, and ^{157}Gd, because they possess a low natural abundance (<40%).

Nevertheless, both the gadolinium and ferric signals can be unambiguously recognized for the Gd^{3+}-ferritin nanoparticles (positioned at g-factors 5.82 and 4.27, respectively). The estimated g-factor difference (≈ 0.05) between the Gd^{3+}-signal of **1** and that of Gd^{3+}-ferritin indicates different spin-orbital interactions and, consequently, a different ligand field/symmetry. Dissociation of Gd^{3+} from GBCA and transfer and binding of Gd^{3+} to the oxyhydroxide core should naturally be accompanied by a change in ligand field/symmetry. The change in g-factor is thus consistent with the proposed formation of Gd^{3+}-ferritin based on the structural analysis of Gd^{3+}-ferritin obtained from HAADF-STEM and SQUID magnetometry.

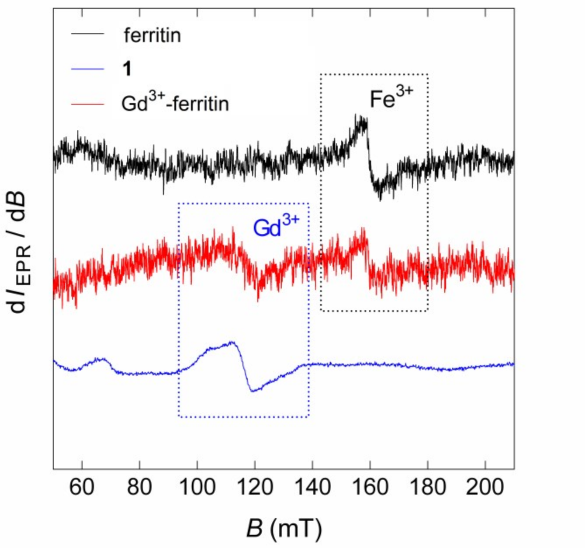

Figure S5. Low-field X-band EPR spectra of ferritin, **1** and Gd^{3+}-ferritin nanoparticles prepared from **1**. All spectra were measured in frozen solutions in HEPES buffer at 77 K and recorded using the same instrumental parameters. Dotted rectangles highlight the characteristic Fe^{3+} and Gd^{3+} signals.

General trends in available kinetic data

Different scientific groups employ different methods to study kinetic inertness, which complicates direct comparison of the inertness of individual GBCAs. However, inspection of available kinetic data obtained under similar conditions revealed some general trends:

1. Complexes with macrocyclic ligands are more inert than those with linear ligands.[35–38]
2. The negatively charged Gd-DOTA complex is more inert than electroneutral macrocyclic complexes with respect to acid-assisted dissociation in acidic solutions.[38,39] However, at neutral pH, all macrocyclic complexes show comparable inertness, even to metal-assisted dissociation. [37,38]
3. The negatively charged Gd-DTPA complex is more inert than electroneutral complexes with DTPA-bis(amide) derivatives,[40] although there are a few exceptions.[41] In neutral solutions, this significant difference in inertness remains.[37,38,42]
4. Complexes of C4-substituted DTPA analogues (BOPTA, EOB-DTPA) are more inert than complexes of DTPA itself. Inertness of EOB-DTPA complexes in neutral solutions approaches those of macrocyclic complexes.[10,41–43]

These general findings are valid *in vivo*[35,43–45] and are fully consistent with the order of lability of complexes studied in this work, which can be viewed as the number of Gd^{3+} atoms present in ferritin nanoparticle after incubation.

References

1 GNU Octave, A high-level interactive language for numerical computations Edition 5 for Octave, 2019.
2 B. Pacakova, S. Kubickova, G. Salas, A. R. Mantlikova, M. Marciello, M. P. Morales, D. Niznansky and J. Vejpravova, Nanoscale, 2017, **9**, 5129–5140.
3 F. Brem, G. Stamm and A. M. Hirt, Journal of Applied Physics, 2006, **99**, 123906.
4 Solution Equilibria Analysis with the OPIUM Computer Program, https://web.natur.cuni.cz/~kyvala/opium.html, (accessed March 7, 2018).
5 R. M. Smith, A. E. Martell and R. J. Motekaitis, NIST Critically Selected Stability Constants of Metal Complexes Database Ver.
6 V. A. Drits, B. A. Sakharov, A. L. Salyn and A. Manceau, Clay Minerals, 1993, **28**, 185–207.
7 Y.-H. Pan, K. Sader, J. J. Powell, A. Bleloch, M. Gass, J. Trinick, A. Warley, A. Li, R. Brydson and A. Brown, Journal of Structural Biology, 2009, **166**, 22–31.
8 K. Kumar, C. A. Chang, L. C. Francesconi, D. D. Dischino, M. F. Malley, J. Z. Gougoutas and M. F. Tweedle, Inorg. Chem., 1994, **33**, 3567–3575.
9 W. P. Cacheris, S. C. Quay and S. M. Rocklage, Magnetic Resonance Imaging, 1990, **8**, 467–481.
10 Z. Baranyai, Z. Pálinkás, F. Uggeri and E. Brücher, European Journal of Inorganic Chemistry, 2010, **2010**, 1948–1956.
11 A. Bianchi, L. Calabi, F. Corana, S. Fontana, P. Losi, A. Maiocchi, L. Paleari and B. Valtancoli, Coordination Chemistry Reviews, 2000, **204**, 309–393.
12 F. Uggeri, S. Aime, P. L. Anelli, M. Botta, M. Brocchetta, C. de Haeen, G. Ermondi, M. Grandi and P. Paoli, Inorg. Chem., 1995, **34**, 633–643.
13 E. T. Clarke and A. E. Martell, Inorganica Chimica Acta, 1991, **190**, 37–46.
14 E. T. Clarke and A. E. Martell, Inorganica chimica acta, 1991, **190**, 27–36.
15 S. Aime, P. L. Anelli, M. Botta, F. Fedeli, M. Grandi, P. Paoli and F. Uggeri, Inorganic Chemistry, 1992, **31**, 2422–2428.
16 A. Bianchi, L. Calabi, C. Giorgi, P. Losi, P. Mariani, P. Paoli, P. Rossi, B. Valtancoli and M. Virtuani, Journal of the Chemical Society, Dalton Transactions, 2000, **0**, 697–705.
17 M. F. Tweedle, J. J. Hagan, K. Kumar, S. Mantha and C. A. Chang, Magnetic resonance imaging, 1991, **9**, 409–415.
18 É. Tóth, R. Király, J. Platzek, B. Radüchel and E. Brücher, Inorganica Chimica Acta, 1996, **249**, 191–199.
19 J. H. Jung, T. W. Eom, Y. P. Lee, J. Y. Rhee and E. H. Choi, Journal of Magnetism and Magnetic Materials, 2011, **323**, 3077–3080.
20 Q. A. Pankhurst, Clays and Clay Minerals, 1992, **40**, 268–272.
21 X. Wang, M. Zhu, L. K. Koopal, W. Li, W. Xu, F. Liu, J. Zhang, Q. Liu, X. Feng and D. L. Sparks, Environ. Sci.: Nano, 2016, **3**, 190–202.

22 Stoner Edmund Clifton and Wohlfarth E. P., Philosophical Transactions of the Royal Society of London. Series A, Mathematical and Physical Sciences, 1948, **240**, 599–642.
23 L. Néel, Advances in Physics, 1955, **4**, 191–243.
24 R. A. Brooks, J. Vymazal, R. B. Goldfarb, J. W. M. Bulte and P. Aisen, Magnetic Resonance in Medicine, 1998, **40**, 227–235.
25 S. Burianova, J. Poltierova Vejpravova, P. Holec, J. Plocek and D. Niznansky, Journal of Applied Physics, 2011, **110**, 073902.
26 M. Bosca, L. Pop, L. Bolundut, N. Tothazan, G. Borodi, I. Vida-Simiti, R. Stefan, A. Popa, E. Culea and P. Pascuta, Ceramics International, 2016, **42**, 1169–1176.
27 S. A. Stoian, C. Paraschiv, N. Kiritsakas, F. Lloret, E. Münck, E. L. Bominaar and M. Andruh, Inorg. Chem., 2010, **49**, 3387–3401.
28 M. Mazur, P. Poprac, M. Valko and C. J. Rhodes, J Sol-Gel Sci Technol, 2016, **79**, 220–227.
29 A. M. Panich, A. I. Shames, N. A. Sergeev, V. Yu. Osipov, A. E. Alexenskiy and A. Ya. Vul', J. Phys. Chem. C, 2016, **120**, 19804–19811.
30 V. Singh, N. Singh, S. Watanabe, T. K. Gundu Rao, M. S. Pathak, A. K. Srivastava, P. K. Singh and S. J. Dhoble, Journal of Electronic Materials, 2017, **46**, 1943–1947.
31 R. Krzyminiewski, Z. Kruczyński, B. Dobosz, A. Zając, A. Mackiewicz, E. Leporowska and S. Folwaczna, Appl Magn Reson, 2011, **40**, 321–330.
32 A. R. Arnold, A. Zhou and J. K. Barton, J. Am. Chem. Soc., 2016, **138**, 11290–11298.
33 P. Kumar, M. Bulk, A. Webb, L. van der Weerd, T. H. Oosterkamp, M. Huber and L. Bossoni, Scientific Reports, 2016, **6**, 38916.
34 J.-M. Gaite, V. V. Izotov, G. R. Bulka, N. M. Khasanova, A. A. Galeev, N. M. Nizamutdinov and V. M. Vinokurov, J. Phys.: Condens. Matter, 1998, **10**, 7609.
35 V. Lawrence, W. Matthai and S. Hartmaier, Investigative Radiology, 1992, **27**, 2.
36 S. Laurent, L. Vander Elst, F. Copoix and R. N. Muller, Investigative Radiology, 2001, **36**, 115.
37 S. Laurent, L. V. Elst and R. N. Muller, Contrast Media Mol Imaging, 2006, **1**, 128–137.
38 M. Port, J.-M. Idée, C. Medina, C. Robic, M. Sabatou and C. Corot, Biometals, 2008, **21**, 469–490.
39 K. Kumar, C. A. Chang and M. F. Tweedle, Inorg. Chem., 1993, **32**, 587–593.
40 L. Sarka, L. Burai and E. Brücher, Chemistry - A European Journal, 2000, **6**, 719–724.
41 S. Laurent, C. Henoumont, L. Vander Elst and R. N. Muller, Eur. J. Inorg. Chem., 2012, **2012**, 1889–1915.
42 Z. Pálinkás, Z. Baranyai, E. Brücher and B. Rózsa, Inorg. Chem., 2011, **50**, 3471–3478.
43 S. K. Morcos, European Journal of Radiology, 2008, **66**, 175–179.
44 W. A. Gibby, K. A. Gibby and W. A. Gibby, Investigative Radiology, 2004, **39**, 138.
45 G. W. White, W. A. Gibby and M. F. Tweedle, Investigative Radiology, 2006, **41**, 272.
